# Catastrophic disassembly of actin filaments via Mical-mediated oxidation

**DOI:** 10.1038/s41467-017-02357-8

**Published:** 2017-12-19

**Authors:** Elena E. Grintsevich, Peng Ge, Michael R. Sawaya, Hunkar Gizem Yesilyurt, Jonathan R. Terman, Z. Hong Zhou, Emil Reisler

**Affiliations:** 1 Department of Chemistry and Biochemistry, University of California (UCLA), Los Angeles, CA 90095 USA; 2 California NanoSystems Institute, UCLA, Los Angeles, CA 90095 USA; 3 Molecular Biology Institute, UCLA, Los Angeles, CA 90095 USA; 4 Departments of Neuroscience and Pharmacology and Neuroscience Graduate Program, The University of Texas Southwestern Medical Center, Dallas, TX 75390 USA; 5 Department of Microbiology, Immunology & Molecular Genetics, UCLA, Los Angeles, CA 90095 USA

## Abstract

Actin filament assembly and disassembly are vital for cell functions. MICAL Redox enzymes are important post-translational effectors of actin that stereo-specifically oxidize actin’s M44 and M47 residues to induce cellular F-actin disassembly. Here we show that Mical-oxidized (Mox) actin can undergo extremely fast (84 subunits/s) disassembly, which depends on F-actin’s nucleotide-bound state. Using near-atomic resolution cryoEM reconstruction and single filament TIRF microscopy we identify two dynamic and structural states of Mox-actin. Modeling actin’s D-loop region based on our 3.9 Å cryoEM reconstruction suggests that oxidation by Mical reorients the side chain of M44 and induces a new intermolecular interaction of actin residue M47 (M47-O-T351). Site-directed mutagenesis reveals that this interaction promotes Mox-actin instability. Moreover, we find that Mical oxidation of actin allows for cofilin-mediated severing even in the presence of inorganic phosphate. Thus, in conjunction with cofilin, Mical oxidation of actin promotes F-actin disassembly independent of the nucleotide-bound state.

## Abstract (web summary)

MICAL Redox enzymes post-translationally modify F-actin to promote its cellular destabilization. Here, the authors present a 3.9Å cryoEM structure of Mical-oxidized F-actin, showing its nucleotide-state dependent dynamic instability and susceptibility to cofilin-induced severing in the presence of inorganic phosphate.

## Introduction

Regulation of actin filament dynamics by post-translational modifications is poorly understood compared to that by non-covalent means, through actin-binding proteins^1^. Selective redox regulation of actin by Mical family enzymes has been found to promote cellular destabilization of F-actin^2^ and play important roles in axonal guidance^3,4^, dendritic organization^5^, synaptic development^6^ and homeostasis^7^, heart^8^ and muscle^6^ development, cell viability^9^, exocytosis^10^, and cytokinesis^11^. Mical stereo-specifically oxidizes F-actin on methionine (M) 44/47 which induces F-actin disassembly^12^—most effectively in conjunction with cofilin^13^. F-actin disassembly can be broadly defined as loss of polymer mass due to depolymerization (monomers’ dissociation from filament ends), which is often facilitated by severing (filaments’ fragmentation resulting in increased number of depolymerizing ends). Most recently, rapid depolymerization of actin filaments upon Mical/NADPH treatment was observed in in vitro assays^11^. However, the molecular basis of such dynamic behavior of Mical-oxidized (Mox) actin and the mechanisms underlying its disassembly are hitherto unknown.

Here, we identify two dynamic states of Mox-F-actin using single filament TIRFM. We show that one of these states undergoes extremely rapid (catastrophic) disassembly (84 subunits/s) in a phosphate/BeFx sensitive manner. In agreement with our TIRFM data, atomic modeling based on the 3.9 Å resolution cryoEM structure of Mox-F-actin resolved two main structural states of Mical-oxidized filaments. One of these structural conformers suggested a new intermolecular interaction that occurs upon Mical oxidation of the actin residue M47 (M47-O-T351). Site-directed mutagenesis indicated that this nascent interaction weakens protomer-protomer contacts to facilitate catastrophic F-actin disassembly. Moreover, we show that oxidation by Mical makes phosphate-rich (“young”) actin susceptible to cofilin severing. Thus, Mical-induced oxidation of actin—including augmentation of cofilin severing—provides a robust mechanism to disassemble different actin forms (ATP/ADP-Pi- and ADP-bound) in response to cellular signaling.

## Results

### Nucleotide-state dependent instability of Mical-oxidized actin

**Fig. 1:**
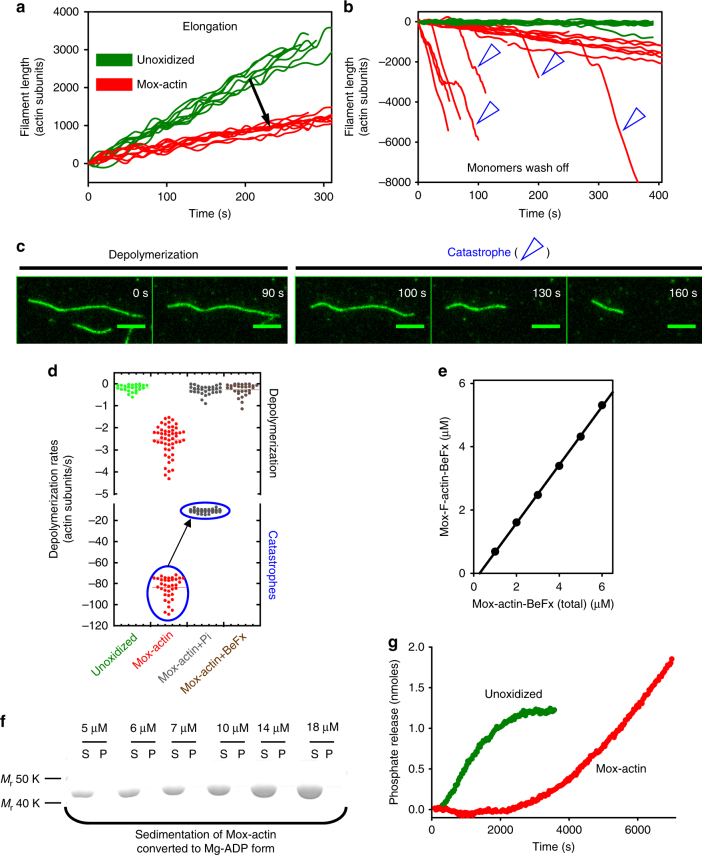
Mical-oxidation of actin induces its nucleotide state-dependent catastrophic disassembly. **a** Mical oxidation of actin inhibits filament elongation. Average elongations rates of unoxidized and Mical-oxidized (Mox) actin filaments (1.24 µM, 15% labeled with Alexa488-SE) were 11.4 ± 0.7 subunits/s (mean ± standard deviation (s.d.); *n* = 10 filaments) and 3.6 ± 0.4 subunits/s (s.d.; *n* = 20 filaments), respectively. **b**, **c** Mox-F-actin undergoes nucleotide state-dependent catastrophic disassembly in the absence of monomers. **b** Mox-actin filaments (red) depolymerize faster than unoxidized F-actin (green) in single filament TIRF assays. Catastrophes are indicated with open blue arrowheads. **c** TIRFM image montages of a single Mox-actin filament switching from depolymerization to the catastrophic disassembly phase. **d** Inorganic phosphate (Pi) reduces the rates of Mox-actin depolymerization and BeFx (mimics ADP-Pi state) abolishes catastrophic disassembly events (encircled in blue). Single filament depolymerization rates were determined from TIRF movies (see Table 1). **e** Mical-oxidized F-actin complex formation with BeFx reduces the critical concentration (Cc) of Mox-actin (0.24 ± 0.05 μM; mean ± s.d.; *n* = 3 independent measurements) compared to that of its mixed nucleotide state (~ 1 μM)^13^. **f** Mical-oxidized ADP-F-actin is highly unstable. Mox-F-actin disassembles when converted to the ADP form. Based on high-speed sedimentation experiments, no ADP-Mox-F-actin was detected in pellets (up to 18 μM concentration); S soluble (G-actin), P pellet (F-actin). *n* = 3 independent experiments. The unprocessed original scan of the gel is shown in Supplementary Fig. 8. **g** Mox-F-actin (red) shows non-saturating kinetics of phosphate release, which is indicative of rapid actin turnover (disassembly and reassembly). Phosphate release from unoxidized actin plateaus as expected (green). Average of three kinetic measurements is shown for each condition

To characterize the dynamic properties of purified Mical-oxidized (Mox) actin, we employed single filament total internal reflection microscopy (TIRFM). We found that elongation of Mox-actin is ~3 fold slower than that of control actin under the same conditions (Fig. 1a, also see Supplementary Fig. 1). Strikingly, monitoring depolymerization of Mical-oxidized actin in the absence of monomers revealed frequent events of extremely rapid disassembly that herein we call catastrophic disassembly or “catastrophes” (Fig. 1b, c, Supplementary Movies 1 and 2). Analysis of our TIRFM data identified two distinct dynamic states in Mical-oxidized actin that were manifested through either fast depolymerization (2.6 ± 0.7 subunits/s) or catastrophic disassembly (84 ± 10 subunits/s) **(**see Fig. 1b–d, Table 1; mean ± s.d., *n* = number of events analyzed). Thus, Mical-mediated oxidation of actin has dramatic effects on F-actin stability^3,12,13^ and prompts its rapid disassembly (also see ref. ^11^).

**Table 1:** Depolymerization of unoxidized and Mical-oxidized F-actin in the absence of actin monomers

Actin form	Actin depolymerization rates (s^−1^)		
	Slow	Fast	
Unmodified F-actin	0.20 ± 0.16 (*n = *24)	**	**Rare events 5.34 ± 1.69 s^−1^ (*n = *9). Rates match ADP-F-actin^14,16^
Mox-F-actin	2.57 ± 0.68 (*n* = 51)	84.26 ± 10.39^#^ (*n = *36)	^#^Catastrophic disassembly
Mox-F-actin + Pi (12.5 mM)	0.28 ± 0.20 (*n = *31)	10.97 ± 1.53 (*n = *43)	Two populations
Mox-F-actin + BeFx	0.27 ± 0.27 (*n = *29)	—	One population

Actin was polymerized and aged on the slides’ surface. Depolymerization of age-matched actin filaments (unmodified and Mical-oxidized) was monitored after 50 min from the beginning of polymerization. Rates are presented as means ± s.d. Number of depolymerization events analyzed is shown in parenthesis

We hypothesized that the observed catastrophic events in Mical-oxidized F-actin disassembly could be linked to its nucleotide-bound state (ATP, ADP-Pi, or ADP). In previous reports, switches in depolymerization rates observed in unmodified actin filaments were attributed to the presence of F-actin stretches with different nucleotide-bound states^14,15^. In our experiments, the fastest depolymerization rates of unmodified F-actin (5.3 ± 1.7 subunits/s) matched closely those previously reported for ADP-F-actin^14,16^ (Table 1). To explore a possible link between the nucleotide-bound state of Mox-actin and the rates of its disassembly, we monitored depolymerization of single Mox-actin filaments formed in the presence of inorganic phosphate (ADP-Pi state) by TIRFM. We found that inorganic phosphate (Pi) dramatically inhibited Mox-actin depolymerization and the rate of catastrophic actin disassembly (Fig. 1d, Supplementary Fig. 2a). However, in the presence of Pi, we still observed two distinct populations of Mox-actin, exhibiting different rates of depolymerization often within the same filament (0.3 ± 0.2 subunits/s and 11 ± 1.5 subunits/s, respectively) (Fig. 1d, Table 1, Supplementary Fig. 2a). We therefore turned to BeFx (which mimics the ADP-Pi bound state in F-actin but binds to it much tighter than Pi). We found that when Mox-F-actin was complexed with BeFx, only a single population of depolymerizing filaments was detected and catastrophic disassembly was completely abolished (Fig. 1d, Table 1, Supplementary Fig. 2a).

To confirm the strong dependence of Mox-actin stability on its nucleotide-bound state we compared the side-by-side critical concentration (Cc) of Mical-oxidized ADP-BeFx- and ADP-bound F-actin (Fig. 1e, f). We polymerized Mox-F-actin for 1 h then converted part of the preparation to the ADP-bound form and stabilized the other part with BeFx (see Methods). Strikingly, no filamentous actin was detected in the ADP-bound Mox-actin preparations under polymerizing conditions (up to 18 µM of total actin) suggesting that it is highly unstable compared to the unmodified ADP-F-actin (compare Fig. 1f and Supplementary Fig. 2b, ref. ^16^). In agreement with our TIRF results, we also found that in the presence of BeFx, Cc of Mox-actin is lower (0.24 ± 0.05 µM) than that of aged Mox-F-actin preparations (Cc~1 µM)^13^, indicating that Mox-actin is most stable in the ADP-Pi-bound state (Fig. 1e).

These experiments also suggest that Mox-F-actin preparations aged overnight (mostly ADP-bound, Cc ~ 1 µM)^13^ are stabilized by a terminal ATP/ADP-Pi cap in the presence of ATP-bound monomers. Therefore, we set out to probe whether substoichiometric amounts of BeFx or/and filament capping affect Mox-actin disassembly. First, employing TIRFM, we tested whether substoichiometric amount of bound BeFx affects depolymerization of Mox-actin (~ 1:6 ratio of BeFx:Mox-actin, based on the Kd ~ 2 µM)^17^. Analysis of fluorescence intensities revealed that binding of substoichiometric amounts of BeFx to Mox-actin inhibits its disassembly (Supplementary Fig. 3a). This result supports the hypothesis that an ADP-Pi cap on the barbed-end can stabilize ADP-bound Mox-actin filaments. Next, we asked whether capping of the barbed end with mouse heterodimeric capping protein (CP) affects Mox-actin disassembly. Strikingly, Mox-actin depolymerization was greatly inhibited (1.7 ± 0.5 subunits/s, *n* = 21 filaments) and catastrophic disassembly was completely abolished (Supplementary Fig. 3b, c). The rates of Mox-actin depolymerization in the presence of CP report on the pointed end depolymerization and appear greater than that of unoxidized actin filaments^16^, which is in good agreement with a previous report^11^. Our results indicate that catastrophic disassembly of Mox-actin filaments predominantly occurs from their barbed ends. Thus, Mox-actin filaments are stabilized in an ATP/ADP-Pi bound state (“young” F-actin), and/or upon barbed end capping.

Next, we validated our TIRFM results by showing that Mical-oxidized actin is also highly dynamic in solution. To this end we monitored phosphate release from Mox-F-actin and unmodified actin filaments. In contrast to unmodified F-actin, we observed non-saturating kinetics of phosphate release from Mical-oxidized filaments which would be consistent with ongoing disassembly and re-polymerization of Mox-F-actin (Fig. 1g). Thus, we documented that ADP-bound Mox-F-actin is dynamically unstable (unless stabilized by an ATP/ADP-Pi cap or/and capping protein) and it is most likely responsible for the catastrophic disassembly events (~ 84 subunits/s) observed by TIRFM (Fig. 1b–d, f; Supplementary Figs. 2 and 3). A second population of Mox-F-actin, depolymerizing at a rate of ~ 2.6 subunits/s (Fig. 1b–d, Table 1), represents a different structural state or/and mixed nucleotide states of Mical-oxidized actin. Moreover, the two dynamic populations of Mox-F-actin observed in the presence of phosphate (Fig. 1d) may indicate the existence of two distinct structural states that bind phosphate with different affinities.

### Structural description of monomeric and filamentous Mox-actin

**Fig. 2:**
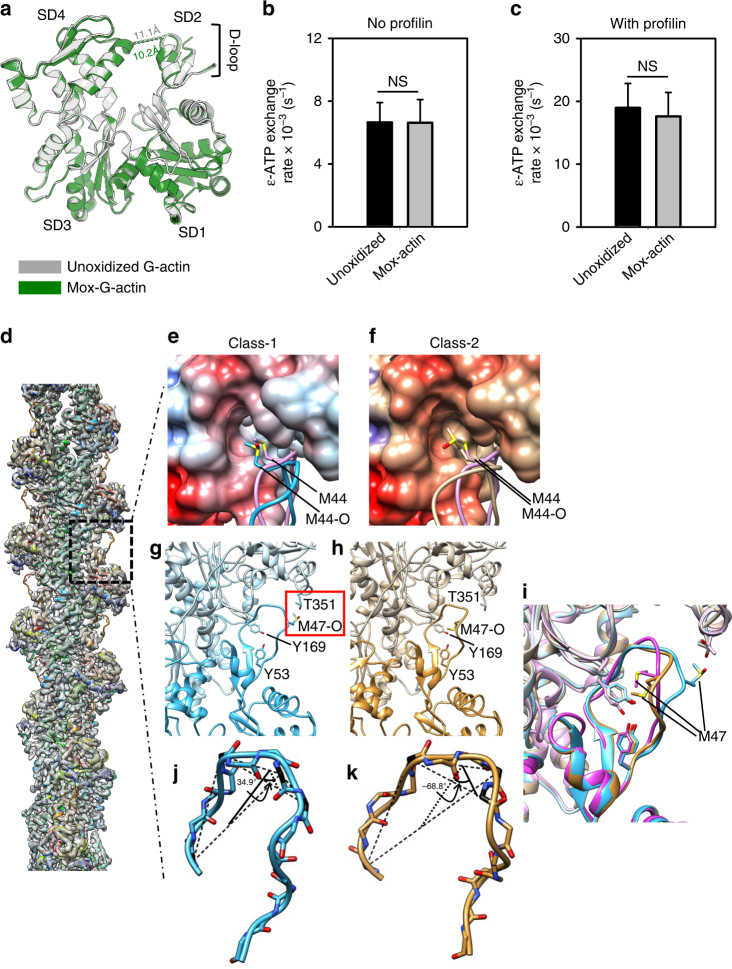
Atomic structures of monomeric and filamentous Mical-oxidized actin. **a** Overlay of the crystal structures of Mical-oxidized (Mox) Ca-ATP-G-actin (2.7 Å resolution, green) and unoxidized (PDB# 1EQY)^18^ G-actin (gray) both in complexes with gelsolin segment-1. Mox-G-actin was crystallized in 7.5% PEG-6000, 150 mM NaCl, 50 mM imidazole, pH 6.6. Width of nucleotide-binding cleft is shown with dashed lines in both structures (10.2 and 11.1 Å). Actin subdomains (SD) 1–4 and the location of the D-loop are indicated. **b**, **c** Rates of nucleotide (ɛ-ATP) exchange in Mical-oxidized and unoxidized G-actin (1 µM). No significant differences in their ɛ-ATP exchange rates were detected either in the absence **b** or presence of 0.1 µM of human profilin **c**. Error bars represent s.d. (*n* = 11 and 10 measurements (with and without profilin, respectively) from 3 independent repeats); NS—not significant (*P* > 0.05), two-tailed Student’s *t*-test. **d**–**k** CryoEM structure of Mox-F-actin at 3.9 Å resolution. 3D structure classification with RELION identified two distinct structural classes (Class-1 and Class-2) **d** Overall cryoEM map of Mox-F-actin computed from all particles (gray shaded surface) is fitted with the atomic model of Class-1 structure (ribbons, rainbow-colored by amino acid sequence). The region boxed with the broken square is shown in zoomed view in (**e**–**i**). **e**, **f** Orientation of the M44 side chain in Class-1 **e** and Class-2 **f** of Mox-F-actin compared to unoxidized (canonical actin model)^19,20^. Oxidation by Mical compromises the geometric surface complementarity between the hydrophobic cleft and the subdomain 2 (SD2) region of actin. **g**–**i** Two different conformations of the D-loop in the atomic models of Class-1 and Class-2 of Mox-F-actin. Zoomed views of the SD2 regions of the atomic models (ribbons) of Classes 1 (cyan, left, **g**) and 2 (tan, middle, **h**). Overlay of the SD2 regions of Classes-1 and 2 of Mox-F-actin and canonical F-actin (pink, right, PDB: 5JLF) is shown in **i**. **j**, **k** Conformational changes in the D-loop of Classes-1 **j** and 2 **k** of Mox-F-actin as measured by a change in the dihedral angle (torsion) between the plane-1 (defined by Cα atoms of residues 39, 43, and 46) and plane-2 (defined by Cα atoms of residues 43, 46, and 47)

To establish the structural basis for the observed behavior of Mical-oxidized actin, we determined the atomic structures of Mical-oxidized actin in non-filamentous and filamentous forms by X-ray crystallography and cryo electron microscopy (cryoEM), respectively. First, we determined whether oxidation by Mical leads to any structural changes in monomeric actin. To this end, we obtained a 2.7 Å resolution crystal structure of Mical-oxidized actin complexed with gelsolin segment-1 (GS1)^18^ (Fig. 2a, Supplementary Table 1). The electron density of the DNAse I binding loop (D-loop)—where Mical’s target M44 and M47 residues reside—is weak, which is indicative of multiple conformations of this loop. In Mox-G-actin structure we observed a slight (~ 1 Å) narrowing of the nucleotide binding cleft (between subdomains 2 and 4) compared to that in the crystal structure of unmodified G-actin-GS1^18^. Narrowing of nucleotide binding cleft could potentially affect nucleotide exchange rate in Mox-actin and affect its recycling in vivo. To test this directly we measured the rates of ɛ-ATP exchange in Mical-oxidized and unmodified G-actin (Fig. 2b, c). We found no statistically significant differences in the nucleotide exchange rates between Mical-oxidized and unoxidized actin in the absence and in the presence of profilin (Fig. 2b, c). Thus, narrowing of the nucleotide binding cleft in the Mox-actin monomer does not affect its nucleotide exchange rates.

Next, we obtained the structure of Mical-oxidized F-actin by cryoEM and helical reconstruction (Fig. 2d–k). We took advantage of the fact that aged Mox-actin retains a filamentous form (Cc ~ 1 µM) despite releasing most of the phosphate (resulting in ADP-bound Mox-actin)^13^. We formed ADP-Mox-F-actin by polymerizing Mical-oxidized ATP-G-actin and then aging resulting filaments for at least two hours to allow for phosphate release. Note, that in this case ADP-Mox-actin is stabilized by an ATP/ADP-Pi cap since ATP and Mox-actin monomers are not removed (Supplementary Fig. 3). We reconstructed a density map of the Mox-actin filament to near atomic resolution, as exemplified by density regions corresponding to actin residues 256–286 (α-helix) and 151–180, 296–302, 326–332 (β-sheet) (Supplementary Fig. 4a–c), However, the D-loop region of this map was disordered. Therefore, we carried out 3D structure classification. This classification yielded two 3D structure classes (49.2% particles in Class-1 and 47.3% particles in Class-2) that differed in the densities of their D-loop regions where Mical’s substrate residues (M44 and M47) are located (Fig. 2e–k, Supplementary Fig. 5). The remaining 3.5% of particles generated a structure with a disordered D-loop. As evident from Supplementary Fig. 5 (right panels), D-loop density of Class 1 is well defined, which enabled reliable atomic modeling of this conformer of Mox-actin (Fig. 2e, g, j). Experimental density of the D-loop region in Class-2 of Mox-actin is indicative of a mixture of two main conformations averaged together. Specifically, comparison of the upper and lower right panels in Supplementary Fig. 5 shows the presence of some particles in the Class-1 conformation (Fig. 2g) in the Class-2 of Mox-actin. Therefore, we modeled the Class-2 density in the D-loop region by excluding the density (Supplementary Fig. 5, right panels) that can be attributed to the Class-1 particles and obtained a model shown in Fig. 2h. Surprisingly, the D-loop conformation of this model is very similar to that of a canonical F-actin structure^19^. The quality of our cryoEM map for both Class-1 and Class-2 is consistent with structural features expected for a 3.9 Å resolution (Supplementary Fig. 4). By a combination of visual inspection of side chain features and the Fourier shell correlation coefficient criteria (both between half-maps and between map and model), the resolutions of both our Mox-actin Class-1 and 2 structures are about 3.9 Å (Supplementary Figs. 4 and 6).

Comparison of the structures of Class-1 and Class-2 of Mox-F-actin to other near atomic resolution F-actin structures^19,20^ revealed that Mical oxidation-induced structural changes in F-actin are confined to the D-loop region where Mical’s substrate residues M44 and M47 are located (Fig. 2d–k, Supplementary Table 2). In both models of Mox-F-actin, the oxidized side chain of M44 (M44-O) still makes contact with the hydrophobic cleft (between subdomains 1 and 3) of actin. However, hydrophobicity of M44 is reduced by its oxidation, which changes the orientation of M44-O side chain most dramatically in Class-1 of Mox-actin. In Class-1, the side chain of M44-O shifts outward (relative to the hydrophobic cleft of actin), weakening intermolecular contacts in Mox-F-actin (Fig. 2e, compare Supplementary Movies 3 and 5). By contrast, in Class-2 model of Mox-actin, the M44-O side chain changes its orientation but still binds tightly to the same cleft, in a manner similar to that in the canonical F-actin (Fig. 2f, compare Supplementary Movies 4 and 5).

Strikingly, our atomic model of Class-1 of Mox-actin suggests that oxidation of M47 allows the formation of a new hydrogen bond between the oxygen on the oxidized sulfur of M47 (M47-O) and the hydroxyl group of T351 (Fig. 2g). Such bonding in Class-1 stabilizes the outward tilt of the C-terminal part of the D-loop (residues 45–51) towards subdomain 1 of the adjacent actin protomer. Such Mical oxidation-induced change in the D-loop conformation can be best described in terms of a rotational twist. Based on our model, the D-loop is twisted in Class-1 as measured by a change in the dihedral angle (torsion) between the plane as defined by Cα atoms of residues 39, 43 and 46, and a second plane defined by residues 43, 46 and 47 (Fig. 2j, k). This dihedral angle differs dramatically in our model of Class-1 of Mox-F-actin (34.9°) from that in Class-2 (−68.9°) and in other high resolution F-actin structures (−22.3° and −81.2° for F-actin-Tm^20^ and ADP-F-actin^21^, respectively). Thus, our cryoEM reconstruction and atomic modeling suggest that Class-1 of Mox-actin exhibits notable differences in the conformation of the D-loop and in the positioning of both the M44-O and M47-O residues.

Our cryoEM results suggest that in contrast to the conformation of Class-1 of Mox-F-actin (where M47-O swung to contact T351) the oxidized M47 likely forms a hydrogen bond with Y169 in Class-2 of Mox-F-actin. This M47-O contact with Y169 is probably responsible for a structural configuration in Class-2 Mox-F-actin similar to that formed via hydrophobic packing among Y169, Y53 and the unoxidized M47 in the Tm-F-actin structure^19,20^. Based on our cryoEM reconstruction, such contact is most likely precluded in Class-1 Mox-actin by the geometry of the D-loop (Fig. 2g–i). In short, our results suggest that the structural state of the D-loop in Class-1 of Mox-F-actin represents a previously unknown conformational state of F-actin, whereas Class-2 is closer to the canonical F-actin structure^19,20^.

### Oxidation of actin’s M47 by Mical promotes F-actin destabilization

**Fig. 3:**
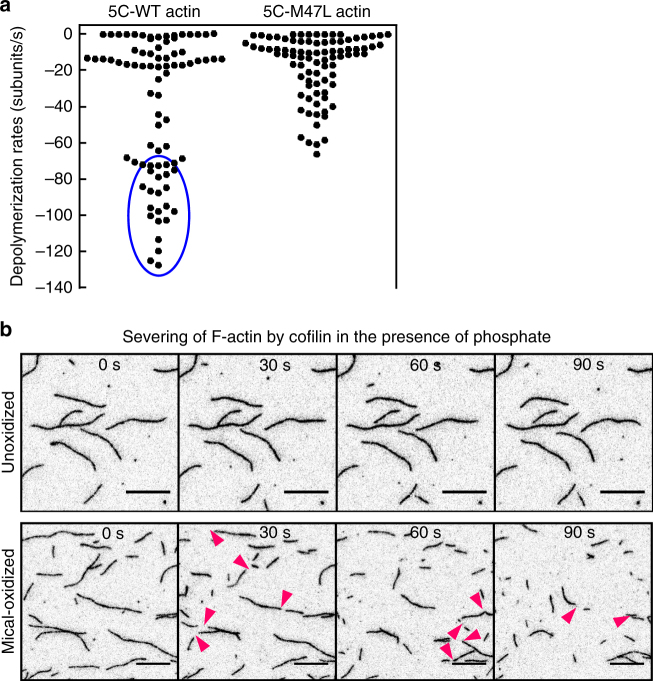
Mical oxidation of both M44 and M47 residues on actin drives its catastrophic disassembly and cofilin severing. **a** Mical oxidation of both—M44 and M47—contributes to F-actin destabilization. Single filament depolymerization rates of 5C-WT and 5C-M47L 2 min after addition of Mical (10 nM) and NADPH (0.1 mM) into the flow chamber. Rates were determined from the TIRF movies (*n* = 3 samples per condition). Two independent preps of each actin form (WT and M47L) were tested. Rates corresponding to the catastrophic disassembly events are encircled in blue. **b** Mox-F-actin in the presence of inorganic phosphate (Pi) (12.5 mM) is rapidly severed and disassembled by cofilin. Cofilin-induced actin disassembly is very slow in unoxidized actin (top panel) and no severing events were observed. Note, that cofilin-mediated disassembly is extensive in ADP-Pi-Mox-F-actin (bottom panel) (also compare to the Supplementary Fig. 2a). Severing events are indicated by magenta arrowheads. Bar = 10 µm

Our TIRF assays and cryoEM reconstruction of Mox-actin revealed the presence of two major structural and dynamic states in Mox-F-actin. We therefore sought to link the structural states of Mox-F-actin (Class-1 or Class-2) to the observed two types of its dynamic behaviors (fast depolymerization and “catastrophes”). Specifically, we wondered whether M47-O-T351 interaction partially compensated for oxidation of M44 and stabilized Mox-F-actin or, alternatively, whether it further destabilized Mox-F-actin by “trapping” the D-loop in an unfavorable conformation. To address this question we employed the previously characterized *Drosophila melanogaster* actin (5C) mutant M47L^12^. In this mutant, Mical oxidation of actin is restricted to M44 such that M47-O-T351 bonding cannot occur. We monitored disassembly of the WT and M47L mutant actins upon on-slide oxidation in single filament TIRFM experiments (Fig. 3a, Supplementary Fig. 7). We observed a wide distribution of depolymerization rates in both WT and M47L actins that probably reflects the contribution of Mical-induced local changes in F-actin stability, which is yet to be assessed. Strikingly, and most importantly, catastrophic disassembly events ≥ 80 subunits/s were detected in Mical-oxidized WT actin but not in oxidized M47L mutants (Fig. 3a). Thus, our results are consistent with the scenario in which oxidation of M47 couples with oxidation of M44 to promote rapid destabilization (“catastrophe”) of Mical-oxidized F-actin.

In addition, our analysis of dynamic behavior of Mical/NADPH-treated non-muscle actin 5C revealed severing events (Supplementary Fig. 7, right panel), as previously observed with skeletal muscle α-actin^12,13^. This is in contrast to a recent report^11^ claiming lack of filaments’ severing/fragmentation upon Mical/NADPH treatment. Although a reason for this discrepancy is not immediately clear, it should be noted that Fremont et al.^11^ did not show if known actin severing proteins (such as cofilin) fragment F-actin under their experimental conditions. In sum, an exact mechanism of Mical-induced F-actin severing is yet to be determined.

These results therefore uncover a molecular basis for the dynamic behavior of Mical-oxidized actin. Furthermore, in light of unique structural and dynamic properties of Mox-actin that we identified, we wondered whether Mical oxidation of actin enables cofilin-mediated disassembly of “young”, ADP-Pi-rich F-actin. It is well established that cofilin has low affinity for ADP-Pi-bound filaments and on its own is inefficient in their disassembly^22,23^. As expected, no cofilin-induced severing was observed with unmodified F-actin in the presence of inorganic phosphate (Fig. 3b, top panel). Dramatically, despite millimolar concentrations of inorganic phosphate we observed extensive severing and disassembly of Mical-oxidized F-actin upon cofilin addition (Fig. 3b, bottom panel). Thus, through its synergy with cofilin, Mical oxidation of actin also provides a unique way to facilitate disassembly of a relatively stable pool of phosphate-rich filaments in response to cellular signaling.

## Discussion

Our results provide key molecular and structural insights into F-actin disassembly by demonstrating that it can proceed catastrophically, in an actin nucleotide state-dependent manner, and by uncovering mechanisms that allow for the dismantling of different forms of actin (ATP/ADP-Pi- and ADP-bound). These observations have come from our characterization of the dynamic and structural states of Mical-oxidized F-actin, and through the demonstration of its nucleotide-state dependent instability (Figs. 1 and 2). In particular, ADP-bound filaments are less stable than ADP-Pi F-actin, which is true for both unoxidized^16^ and Mical-oxidized F-actin. Phosphate binding restricts intramolecular motions of individual actin protomers and stabilizes filaments’ structure^24,25^. Likewise, phosphate release increases overall F-actin dynamics, especially that of its D-loop^23,25^. It has been documented that in ADP-F-actin, the D-loop visits multiple conformational states, including extreme ones which, if captured by cross-linking, can be incompatible with F-actin structure^26^. We propose that Mical oxidation of a key M44 residue (which is necessary for in vivo and in vitro Mical effects)^12^ takes such “dynamization” of ADP-F-actin to the extreme by weakening protomer-protomer contacts and allowing the D-loop to explore a wider range of infrequent, potentially filament-destabilizing conformations^26^. This allows for the formation of the M47-O-T351 contact, which captures one of the extreme D-loop conformers in Class-1 of Mox-actin and promotes F-actin destabilization (Fig. 2g–i). Indeed, based on our mutational analysis, disruption of the M47-O-T351 contact in the Mical-oxidized M47L actin mutant abolishes its catastrophic (> 80 subunits/s) disassembly (Fig. 3a). Thus, our data supports the hypothesis that Mical oxidation of both M44 and M47 residues of actin contributes to the rapid destabilization of filaments.

Our data also provide important insights into the role of the D-loop in F-actin stability. In particular, our atomic modeling predicts that by “trapping” of the extreme rotational twist of the D-loop, the M47-O-T351 interaction in Class-1 Mox-actin disrupts the M47-Y169 contact that occurs in the F-actin-Tm complex^19,20^. Moreover, M47-Y169 contact is also missing in 4.7 Å cryo-EM-based model of unoxidized ADP-F-actin^21^. This loss of the M47-Y169 interaction might be characteristic for less stable forms of actin (Mox-F-actin Class-1 as well as unstabilized ADP-F-actin^21^) compared to the stabilized F-actin-Tm complex^19,20^. We therefore speculate that the M47-Y169 contact may be important for maintaining the D-loop conformation in complementary shape to the hydrophobic cleft between actin subunits in which it binds^19^. Moreover, a hydrogen bond between M47 and F352 was modeled based on a 4.7 Å resolution ADP-F-actin structure^21^. This interaction may capture a less stable conformer of the D-loop in unoxidized ADP-F-actin and contribute to the fast depolymerization rates (~ 6 subunits/s) measured for this actin form in single filament TIRF assays (Table 1 and refs. ^14,16^).

During cellular events, such as the collapse of axonal growth cones, different actin forms (ATP/ADP-Pi and ADP) need to be disassembled rapidly. How this is achieved is not immediately clear since the best characterized actin disassembly factor—cofilin—has low affinity to ADP-Pi F-actin^22,23^. We previously showed that Mical oxidation of actin dramatically increases the efficiency of cofilin-induced disassembly of ADP-bound Mox-F-actin (aged) and its copolymers with unmodified actin^13^. Here we have found that Mical oxidation of actin allows for extensive cofilin severing of ADP-Pi bound filaments. We propose that enhanced cofilin binding to Mox-actin improves its severing, which exposes fast depolymerizing (ADP-Pi bound) and dynamically unstable (ADP bound) Mox-actin regions (Fig. 1d), thereby dramatically amplifying F-actin disassembly. Thus, oxidation by Mical provides a unique way to disassemble different forms of F-actin in response to cell signaling.

## Methods

### Protein purification

Rabbit skeletal actin (RSA) was purified from acetone powder^27^. Recombinant Drosophila 5C actins (WT and M47L mutant) were purified from baculovirus-infected Sf9 cells^12^. Recombinant human profilin-1 was expressed in *E. coli* and purified using poly-L-proline affinity column^28^. Recombinant gelsolin segment-1 (6His-GS1) was expressed in *E. coli* and purified using metal chelate chromatography (Ni-NTA, Qiagen)^26^. Human cofilin-1^29^ was expressed in *E. coli* BL21(DE3)pLysS cells and purified using SP Sepharose FF (GE Healthcare) followed by gel filtration on HiLoad 16/60 Superdex 75 (Amersham Biosciences). Drosophila Mical^redoxCH^ construct (referred to as Mical in this study) was expressed in *E. coli* ArcticExpress cells (Stratagene) and purified by two rounds of Ni^2+^ affinity chromatography followed by anion exchange on HiTrap Q-FF (GE Healthcare)^30^. *Acanthamoeba castellanii* actin (AA) and mouse heterodimeric capping protein (CP) were a kind gift from Dr. Margot Quinlan (UCLA).

### Preparation of actin forms

RSA was labeled with Alexa488-succinimidyl ester (SE) using standard approach^13^ that included: (1) actin polymerization with 100 mM KCl; (2) high speed pelleting (TLA110 rotor at 60,000 rpm for 30 min at 4 °C); (3) dialysis (3 h) against labeling buffer (50 mM PIPES, pH 6.8, 50 mM KCl, 0.2 mM CaCl_2_, 0.2 mM ATP); (4) overnight incubation at 4 °C with three-fold molar excess of Alexa488SE dye. The labeling reaction was stopped by addition of 1–2 mM DTT followed by high speed pelleting, actin depolymerization on dialysis (GB_2_: 2 mM Tris, pH 8, 0.2 mM CaCl_2_, 0.2 mM ATP, 0.5–1 mM DTT), and gel-filtration (Superdex S200 10/300 GL). RSA labeling with pyrene maleimide was carried out in thiol-free GB_2_ supplemented with 2 mM MgCl_2_ and 100 mM KCl at 1:2.5 (actin:dye) molar ratio for 1 h on ice. The resulting pyrene-labeled F-actin was pelleted, depolymerized (GB_2_), and gel-filtered on Superdex S200 16/60 column. Mical-oxidized RSA (Mox-actin) was prepared and purified according to the published protocol^13^ with minor modifications. In brief, unlabeled RSA was oxidized on M44 and M47 at 1:50 (Mical:actin) ratio in the presence of 0.2 mM NADPH, for 2 h at room temperature (RT), unless stated otherwise. After 2 h actin was centrifuged at 100,000 g for 20 min at 4 °C. The resulting supernatant containing Mox-actin was dialyzed overnight against GB_2_ then gel-filtered on Superdex S200 16/60 column. Pyrene- and Alexa488-SE-labeled actin forms were oxidized by Mical in GB_2_ at 70:1 (actin:Mical) molar ratio in the presence of 100 μM NADPH for 1 h at RT. The resulting actin was dialyzed overnight against GB_2_ then centrifuged (TLA100 rotor, 90,000 rpm, 30 min, 4 °C). Mg-ADP-F-actin was prepared by incubation of Mg-ATP-F-actin with 1 mM dextrose and hexokinase (8 U/ml of actin) for 1 h on ice^31^. BeFx-F-actin complexes were obtained by mixing Mg-F-actin with 0.1 mM BeCl_2_ and 5 mM NaF (1× BeFx) followed by 1 h incubation on ice^31^.

*Acanthamoeba castellanii* actin (AA) was labeled with Cy3b as follows. Thiol-free AA was polymerized for 1 h at room temperature (RT) with 2 mM MgCl_2_ and 50 mM KCl. Cy3b dye was added to AA F-actin at 1:3 (AA:Cy3b). Labeling was carried out for 1 h on ice. To stop the labeling the reaction was supplemented with 1 mM DTT and 10 mM Hepes (pH 7) and incubated for 15 min at RT. AA-Cy3b was pelleted in TLA110 rotor at 80,000 rpm for 30 min at 4 °C and recovered in supernatants. Excess of label was removed by dialysis against GB_2_ containing 5 mM β-marcaptoethanol (instead of DTT) followed by Sephadex G-50 spin column. Efficiency of labeling was ≥ 90%.

### Critical concentration determination

Mg-ATP-actin (Mical-oxidized and unoxidized) was polymerized in 1xKMEH7.4 buffer (50 mM KCl, 2 mM MgCl_2_, 0.2 mM EGTA, 10 mM HEPES, 0.2 mM ATP, 0.5 mM DTT, pH 7.4) for 1 h at RT. This actin preparation was used to generate BeFx-F-actin and ADP-F-actin for side-by-side experiments as described in the main text. Critical concentrations (Cc) of ADP- and BeFx F-actin were determined using standard protocol^13^. In brief, actins were diluted into 1xKMEH7.4 buffer supplemented either with 0.5 mM ADP instead of ATP (for ADP-F-actin samples) or with 1× BeFx (for BeFx-bound samples) followed by 4 °C overnight incubation. Supernatants and pellets were separated by high speed centrifugation (TLA100, 62 K, 30 min, 4 °C) and analyzed by SDS-PAGE. Gels were stained with Coomassie Blue and densitometry was performed using Scion Image software. The intersects of the linear plots of pelleted actin vs. total actin with the abscissa yielded Cc.

### Nucleotide exchange

Ca-ATP-G-actin was incubated with 10 fold molar excess of ɛ-ATP for 2 h on ice in ATP-free GB_2_ buffer (2 mM Tris, 0.2 mM CaCl_2_, 0.5 mM DTT, pH 8). Excess of ɛ-ATP was removed using Sephadex G-50 spin columns. Actin preparations were supplemented with 5 µM ɛ-ATP. Nucleotide exchange in Mg-ATP-G-actin (1 µM) or Ca-ATP-G-actin (in the presence of profilin) was monitored after the addition of 100 µM of ATP. Excitation and emission wavelengths were 350 nm and 400 nm, respectively. The resulting curves were fitted with single exponential decay function using SigmaPlot software.

### Phosphate release

Phosphate release was assessed using EnzChek phosphate assay kit (Invitrogen, E6646) according to the manufacturer’s instructions with the following modifications. Reaction mixtures were supplemented with additional 1 mM MgCl_2_ (2 mM final concentration), 50 mM KCl, and 0.2 mM ATP to match closely the conditions of other experiments. Ca-ATP-G-actin (Mical-oxidized or unoxidized) (tube #1) and all other components of the reaction (tube #2) were pre-incubated separately for 10 min at room temperature and mixed together to start actin polymerization. Phosphate release upon actin polymerization was monitored by increase in absorbance at 360 nm.

### TIRF microscopy

Untethered actin filaments were imaged on Pluronic coated surface^32^. Flow chambers (V~12 μl) were assembled using a single layer of permanent double-sided Scotch tape. For each sample the flow chamber was treated with 2 chamber volumes (CV) of 1% Pluronic F127 solution (Sigma, P2443) for 3 min then equilibrated with 2 CV of 1xTIRF imaging buffer (10 mM HEPES, 2 mM MgCl_2_, 50 mM KCl, 0.2 mM EGTA (pH 7.4) supplemented with 50 mM DTT, 0.2 mM ATP, 20 mM glucose, 0.5% methyl cellulose). G-actin mixtures (15% Alexa488-SE labeled) were incubated for 3 min at RT with Mg/EGTA exchange buffer (0.2 mM EGTA, 50 μM MgCl_2_), mixed with the 2xTIRF imaging buffer, and the resulting mixture (4 CV) was introduced into the flow chamber. Actin mixtures and all the subsequent washes were supplemented with 0.05 mg/ml casein, 0.25 mg/ml glucose oxidase, 50 μM catalase to minimize radical damage and photobleaching during imaging. For depolymerization experiments RSA and Mical-oxidized F-actin (0.4–2.5 µM) were formed in flow chambers and aged on the surface for at least 45 min. During on-slide polymerization, slides were kept in sealed secondary containers to minimize the drying. Unpolymerized actin monomers were washed off with 2 CV of 1xTIRF imaging buffer and depolymerization movies were recorded. When indicated, actin was polymerized in (and washed with) 1xTIRF imaging buffer supplemented with one of the following: phosphate (Pi) (12.5 mM), Na_2_SO_4_ (12.5 mM) or 1×BeFx (0.1 mM BeCl_2_ and 5 mM NaF). For the experiments with substoichiometric amounts of BeFx we were using 400-fold dilution of 1×BeFx stock (0.25 µM final) maintaining the concentration of NaF at 5 mM. The amount of BeFx incorporated into Mox-actin filaments under subsaturating conditions was estimated using reported Kd value of BeFx to F-actin (~ 2 µM) and stoichiometry 1:1^17^. In the experiments involving mouse heterodimeric capping protein (CP), Mox-actin was first polymerized in flow chambers without a capper followed by the simultaneous removal of Mox-actin monomers and introduction of CP (50 nM). To test for cofilin-induced severing of ADP-Pi-F-actin, unoxidized or Mox-actin was polymerized in the presence of Pi (as above) then washed with 1xTIRF imaging buffer supplemented with 12.5 mM Pi and 150 nM human cofilin-1.

For on-slide Mical oxidation of actin, unoxidized 5C Drosophila F-actin was formed in flow chambers as described above. Since Drosophila 5C-actin contains a total of 7 cysteines, which complicate site-specific labeling, we used 7.5% of Cy3b-maleimide labeled cytoplasmic actin from *Acathamoeba castellanii* as a fluorescent reporter. To start the oxidation, mixtures of Mical (10 nM) and NADPH (0.1 mM) in 1xTIRF imaging buffer were added to the flow chamber. Oxidation was allowed for 2 min then depolymerization movies were recorded. All TIRF data was analyzed using ImageJ (Fiji) software (NIH, Bethesda, MD).

### Cryo electron microscopy and reconstruction

For cryo electron microscopy (cryoEM) samples RSA was oxidized at 1:10 (Mical:actin) ratio in the presence of 0.4 mM NADPH and purified as described above (also see ref. ^13^). Mical-oxidized actin was polymerized for at least 2 h in the following buffer: 5 mM Tris, 0.2 mM CaCl_2_, 0.2 mM EGTA, 2 mM MgCl_2_, 50 mM KCl, 1 mM DTT, 0.3 mM ATP (pH 7.4), and imaged at 6 µM concentration.

Aliquots of 2.5 µL of polymerized Mox-F-actin were applied onto a “baked”^33^ Quantifoil 1.2/1.3 μm, 200 mesh grid, blotted for 4.5 s at force 1, then flash-frozen in liquid nitrogen cooled liquid ethane in a Vitrobot Mark IV (FEI). CryoEM data were collected in an FEI Titan Krios microscope (operated at 300 kV) equipped with a Gatan imaging filter (GIF) (slit width 20 eV) and K2 Summit direct electron camera in counting mode using Leginon software package^34^ for automation. Defocus values were controlled with Leginon by applying a single 3.0 μm target defocus. Dose-fractionation movies were recorded at a frame rate of 5 Hz with a 10 s shutter time. The total accumulated dosage was 60 e/A^−2^, with each frame 1.2 e/A^−2^ and a temporal dose rate of 6 e/A^−2^s (measured on Digital Micrograph (Gatan) software).

Frames were aligned according to Li et al.^35^ except that an iterative alignment scheme was employed^36^. We used the summary of all frames to determine defocus and particle locations, and 3rd–20th frames for data processing. The defocus parameters of the data were determined by CTFFIND3^37^. We selectively included images within a defocus range of 1.5–5 μm.

Actin filaments were selected manually in EMAN^38^ helixboxer. A total of 68,168 filament regions were selected. These filaments were segmented into 237,652 boxes of 384 × 384 pixels, in which each box progresses 38 pixels along the helical axis (90% overlap). These boxes were subjected to 2D classifications as prescribed in Relion manual^39^ to eliminate bad particles, yielding 208,364 boxes to the 3D classification step. We used a 3D classification (Relion with an implementation of IHRSR^40,41^) with three classes initially and found that one class was mainly containing bad particles (3.5% occupancy). The other two classes (49.2% and 47.3%, respectively) were slightly different as we pushed the working resolution down to about 8 Å by refining the angular sampling. 3D auto-refinement in Relion/IHRSR of the boxes attributed to the two classes (102,762 and 102,015 boxes, respectively) generated two volumes with notable difference limited to the subdomain 2. The resolution of the two volumes were similar, both estimated at 3.9 Å with a consideration of “gold-standard” Fourier-shell correlation plot (0.143 criteria), ResMap^42^ and the matching between side chain densities and their models.

A further experiment was done to confirm the classification findings with a 3D classification of 7 classes. Only two classes out of the seven showed enough resolution (to see secondary structural elements in the map) when refined in the same way above, suggesting that there were only two major conformational populations in the dataset.

### Atomic modeling of Mox-F-actin

Atomic models were built with Coot^43^. We docked a canonical actin model (PDB 5JLF^20^) to each of our density maps. We found that all parts of it, except for subdomain 2, were within convergence radii of real space refinement software (see below) into our density map. We therefore solely remodeled subdomain 2 in Coot and kept the rest of the canonical model in a reliance for the refinement software to bring the model into a good fit. We then carried out model refinement for each of the modified canonical model with the phenix.real_space_refine command of the Phenix package^44^ using default settings.

### Mox-G-actin structure determination

Mical-oxidized Ca-G-actin-GS1 complex was crystallized under conditions close to those reported by McLaughlin et al.^18^. Specifically, proteins were mixed in 1:1 molar ratio (final concentration of the complex was 8.2 mg/ml) in GB_2_ buffer supplemented with 0.2 mM ATP, 0.25 mM TCEP, 1 mM PMSF and 0.02% NaN_3_. The ratio of protein complex to precipitant was 1:1 (v/v). Volumes of hanging drops and reservoir solutions were 2 µl and 500 µl, respectively. The largest crystals (plates) grew overnight in 7.5% PEG-6000, 150 mM NaCl, 50 mM imidazole, pH 6.6. Smaller crystals were also obtained when concentration of PEG600 was increased to 10%.

Data collection and reduction proceeded as follows. The crystals were cryo-protected by quickly dipping them in solution of 65% reservoir and 35% methylpentanediol (v/v), and then immediately cryo-cooled to 100 K in a nitrogen gas stream. Diffraction data were collected at the Northeastern Collaborative Access Team beamline, 24-ID-C, at the Advanced Photon Source at Argonne National Laboratory. Data were recorded from two crystals on a Pilatus 6 M detector using a wavelength of 0.9795 Å, detector distances of 250, 400, and 500 mm, 0.5 s exposures, and oscillation angle of 0.5°. A total of 360° of data were collected from one crystal and merged with two runs from a second crystal, spanning 360° and 350° of data, respectively. The merged data extends to a resolution of 2.4 Å, with a CC_1/2_ of 0.83, I/σ = 2.3 and in *R*_merge_ = 171% the 2.45–2.40 Å resolution shell. Overall, the data multiplicity was over 38-fold. Using the more conservative *R*_merge_ statistic, the resolution limit of the data would be drawn at 2.75 Å which reached 52.8% in the shell 2.91–2.75 Å resolution shell, (I/σ = 5.2). This more conservative limit is reported in Supplementary Table 1.

Structure determination and refinement were performed as follows. The starting model for refinement was the crystal structure of reduced actin-gelsolin, PDB ID code 1EQY^18^. The crystal was isomorphous to the Mical-oxided actin-gelsolin complex; therefore, no molecular replacement was necessary. Refinement was performed using the program Buster^45^. The model was manually adjusted to fit the maps using the program Coot^43^. The model was refined using all data to 2.4 Å resolution. Refinement statistics are reported in Supplementary Table 1 to the more conservative limit of 2.75 Å. The final model was validated using the program PROCHECK^46^ which revealed 92.4% of residues are in the most favored regions of the Ramachandran plot, 7.4% of residues are in additional allowed regions, and one residue is in a generously allowed region. There were no Ramachandran outliers. The final model had an Errat score of 96.8%^47^. The structure was illustrated using the program Pymol.

For structural comparison, the unmodified and Mical-oxidized actin-gelsolin complexes were superimposed on subdomains 1, 3, and 4 using the secondary structure matching algorithm^48^ implemented in Coot^43^.

### Statistics and reproducibility

All TIRF microscopy and solution experiments were repeated at least two separate independent times and there were no limitations in repeatability. At least two independent protein purifications and multiple independent actin biochemical experiments were performed with similar results. The figure legends list the sample size for each experiment. To the best of our knowledge the statistical tests are justified as appropriate. No cell lines were used in this study.

### Data availability

The authors declare that all data supporting the findings of this study are available within the article and its Supplementary Information files or from the corresponding authors upon reasonable request. The crystal structure of Mical oxidized monomeric actin with gelsolin segment-1 has been deposited in the protein databank under accession code: PDB ID: 5UBO. The atomic models and the cryoEM density maps for Classes 1 and 2 of Mox-F-actin have been deposited in the Protein Data Bank and EMDB databases under the accession codes: 6AV9, 6AVB and EMD-7007, EMD-7008, respectively.

## Electronic supplementary material

Supplementary Movie 1

Supplementary Movie 2

Supplementary Movie 3

Supplementary Movie 4

Supplementary Movie 5

Supplementary Information

Description of Additional Supplementary Files
