# Supplementary material for: Catastrophic disassembly of actin filaments via Mical-mediated oxidation
Source: Nat Commun. 2017 Dec 19;8:2183. doi: 10.1038/s41467-017-02357-8 (PMC5736627; doi:10.1038/s41467-017-02357-8)
Supplement: Supplementary file 6 — Supplementary Information [file 41467_2017_2357_MOESM6_ESM.pdf]

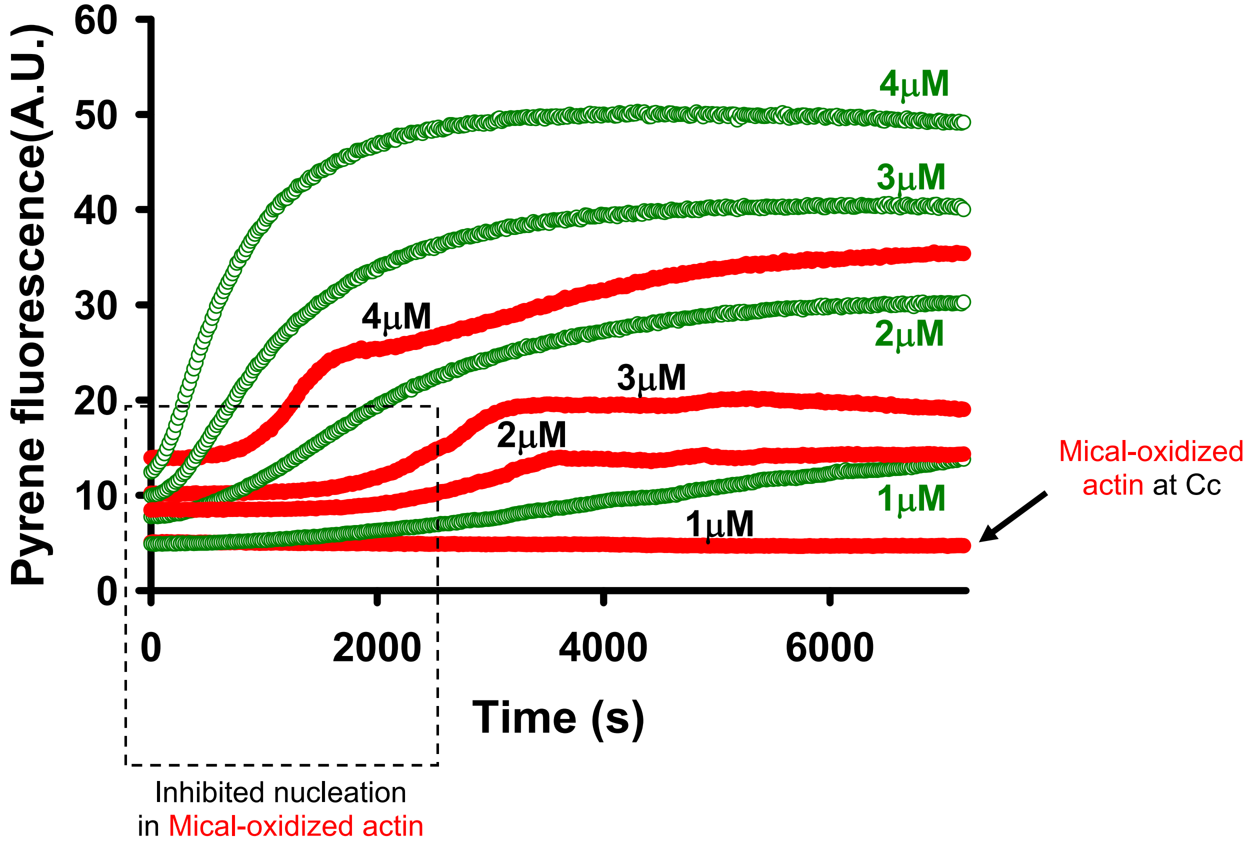

Supplementary Figure 1. **Polymerization kinetics of Mox-actin compared to unoxidized actin**. Polymerization was monitored using 5% of pyrene-maleimide actin (Mox- or unoxidized). Note the greater lag phase in Mox-actin containing samples (red) compared to the unoxidized actin (green) due to inhibited nucleation. As expected, no polymerization was observed under our experimental conditions at 1μM of Mox-actin (concentration ≤ critical concentration (Cc))[1].

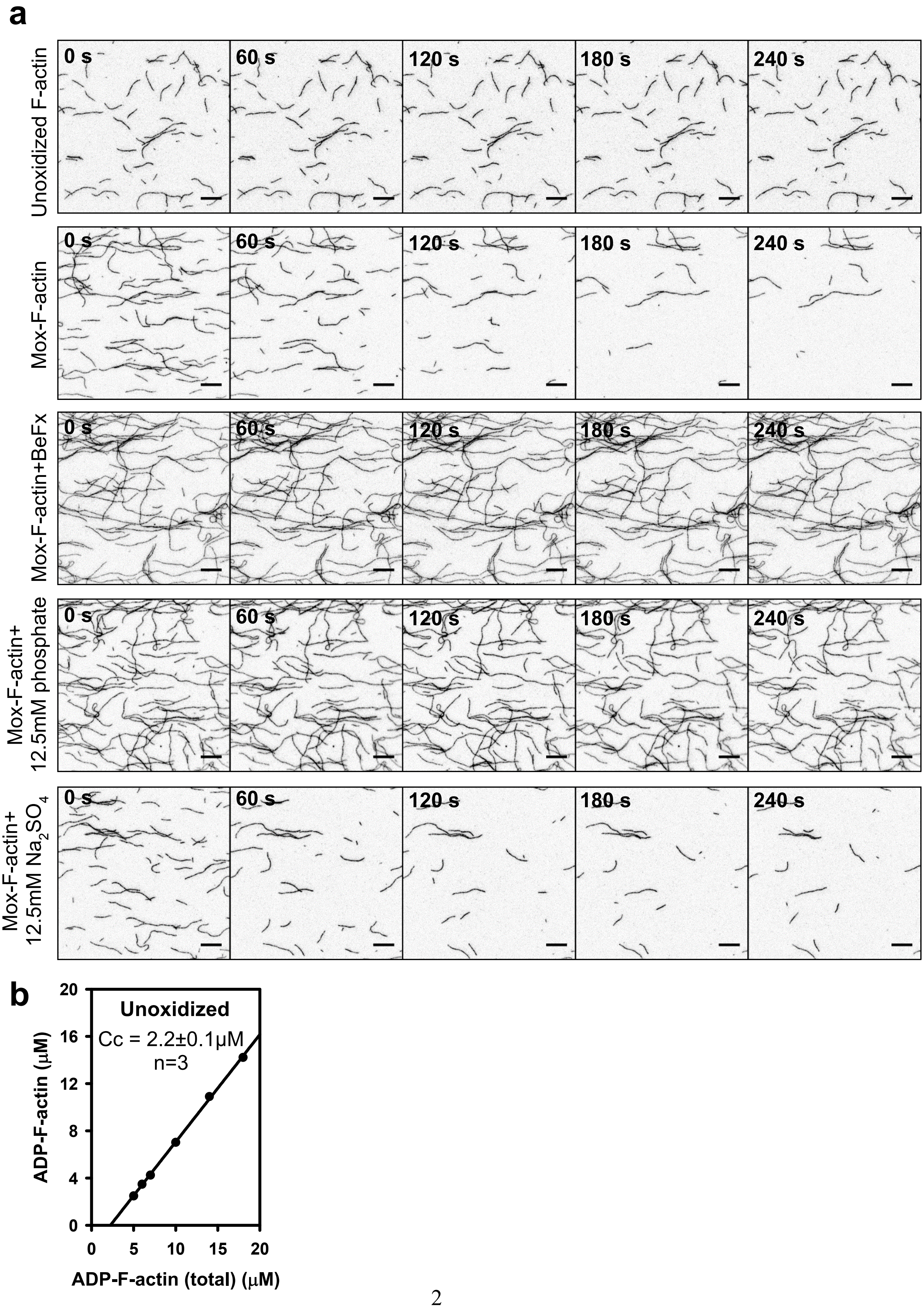

a
Unoxidized F-actin
Mox-F-actin
Mox-F-actin+BeFx
Mox-F-actin+
12.5mM phosphate
Mox-F-actin+
12.5mM Na_2SO_4
0 s
60 s
120 s
180 s
240 s
b
Unoxidized
Cc = 2.2±0.1μM
n=3
ADP-F-actin (μM)
ADP-F-actin (total) (μM)
0
4
8
12
16
20
0
5
10
15
20

Supplementary Figure 2. **(a) Depolymerization of different forms of filamentous actin upon removal of monomers**. Unoxidized (0.4µM) (top panel) and Mox-actin (1.5µM) were polymerized on the slide surface as described in **Methods,** then residual monomers were washed off to assess depolymerization rates. Note the substantial loss of filaments' mass over time in Mox-F-actin due to catastrophic disassembly events (quantified in **Fig. 1d**). Depolymerization of Mox-F-actin is substantially slower in the presence of inorganic phosphate and BeFx (mimics ADP-Pi bound F-actin state) but not upon the increase of ionic strength with Na_2SO_4 (control for phosphate-containing samples). Note that unoxidized actin filaments are shorter due to the different starting concentration of actin. Montages were made from representative TIRF movies (also compare **Movies 1** and **2**). Bar=10µm. **(b) Critical concentration of unoxidized ADP-F-actin (control)**. Note that under the same conditions, Cc of Mox-ADP-F-actin is > 18µM (**Fig 1f**). Cc ± s.d. (n=3 independent preparations of ADP-F-actin). Representative data set is shown.

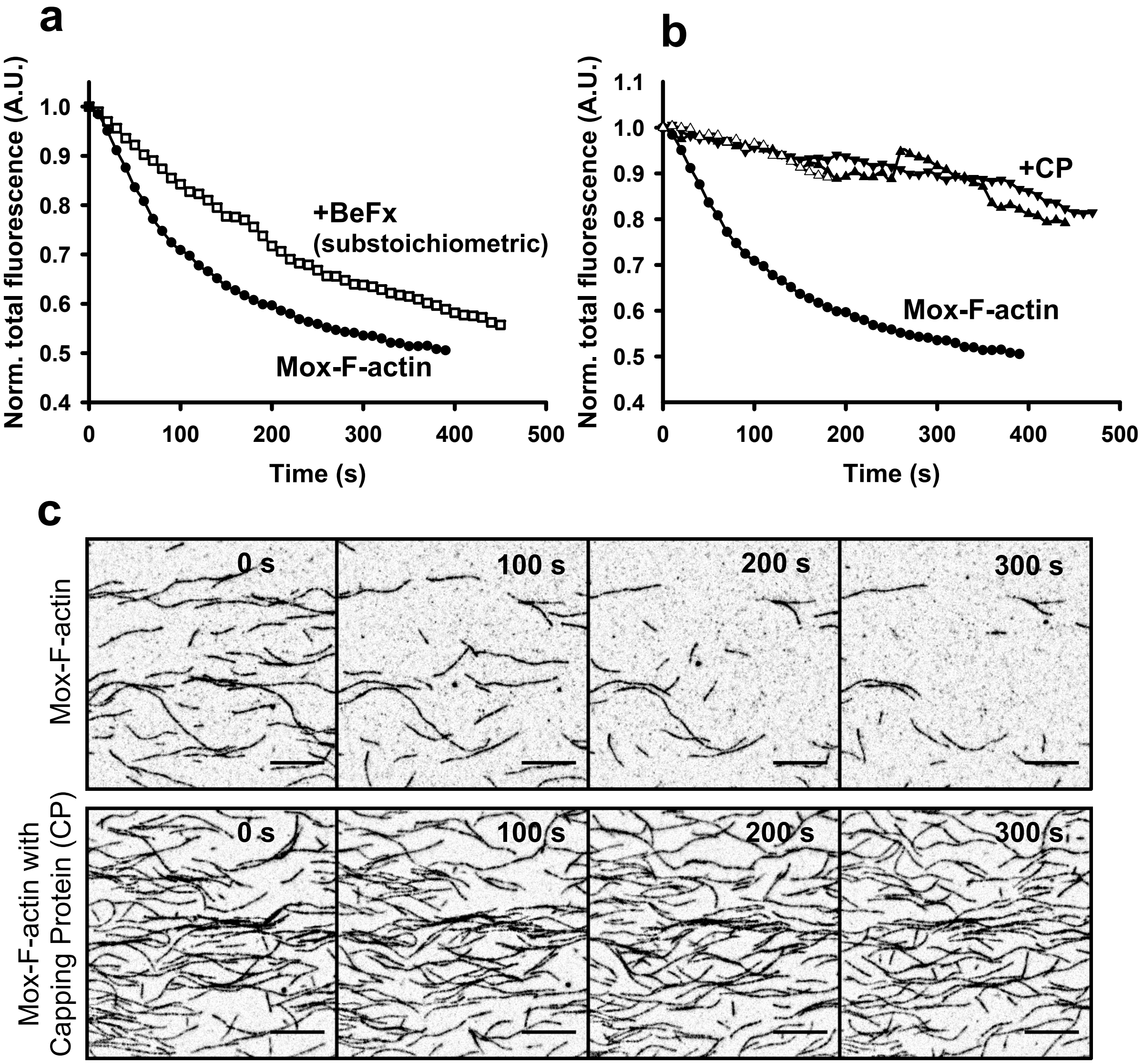

Supplementary Figure 3. **Substoichiometric amounts of BeFx and barbed end capping stabilize Mox-actin filaments**. **(a)** Inhibition of Mox-actin depolymerization by substoichiometric amount of BeFx. Mox-actin (1.5 μM, 15% Alexa488-SE) was polymerized in the absence and presence of 0.25 μM BeFx and 5mM of NaF. Depolymerization was induced by washing off Mox-actin monomers with BeFx-free buffer. Total fluorescence intensity per field of view was measured using ImageJ software. Averages of the fluorescence intensities from the representative movies are shown (Mox-actin *(black circles)*: n=3 movies; Mox-actin-BeFx *(open squares)*: n=2 movies). **(b-c)** Inhibition of Mox-F-actin disassembly by capping of the barbed end. Depolymerization of Mox-actin was induced by washing off the monomers with the buffer containing none *(black circles)* or 50 nM of mouse heterodimeric protein (CP) *(triangles)*, n=3 samples/conditions. Three sets of data obtained in the presence of CP are shown in (b) *(triangles)*. Average of the 3 samples is shown for Mox-F-actin alone *(black circles)*. **(c)** Time point images from representative TIRF movies show that depolymerization of Mox-F-actin is substantially slower in the presence of CP *(lower panel)* than Mox-F-actin alone *(top panel)*. Scale bar=10 μm

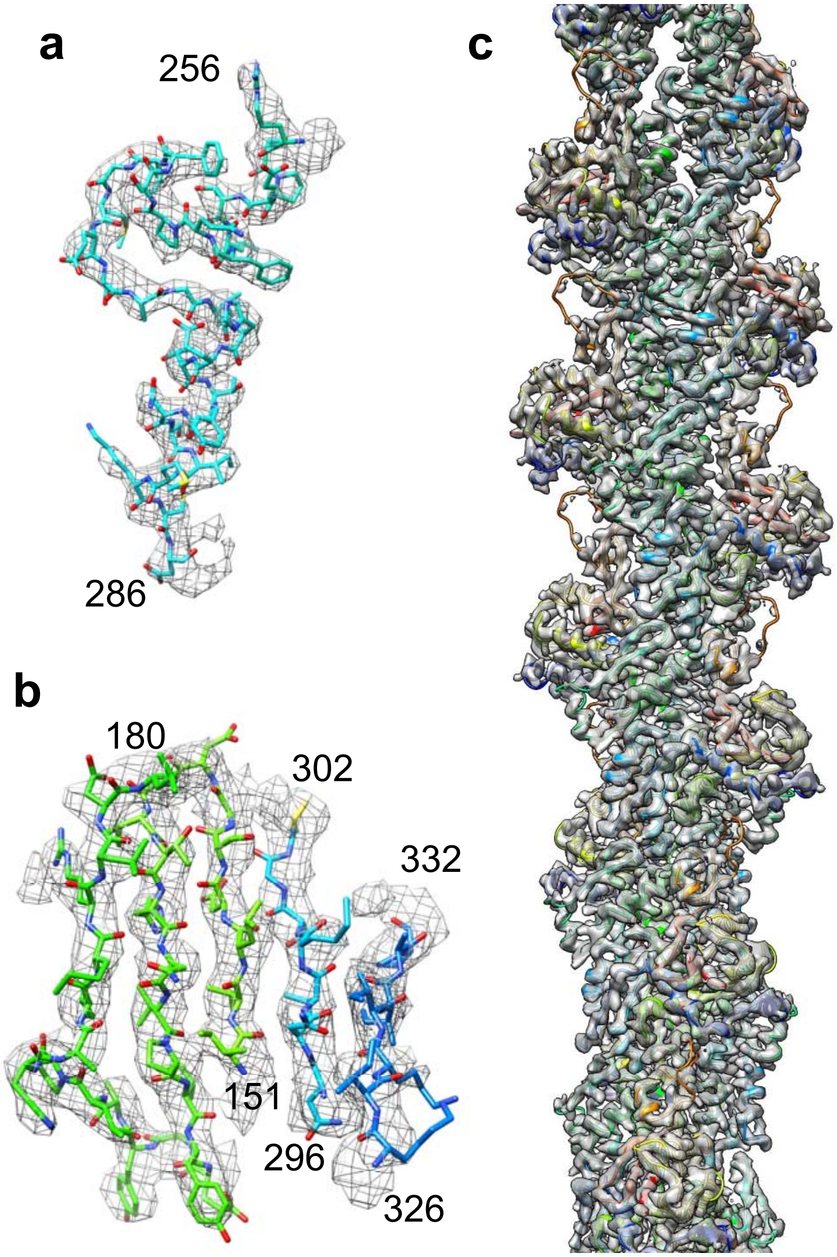

Supplementary Figure 4. **Quality of cryoEM reconstructions for Mox-F-actin**. **(a-c)** CryoEM map calculated from all particles without classification. **(a-b)** An α-helical region **(a)** and a β-sheet region **(b)** of the density map (wireframe) superimposed with their atomic models (sticks, rainbow colors). In both panels, the amino acid side chain features match their models. **(c)** The overview of the density map rendered in shaded surfaces superimposed with atomic models for its subunits (ribbon, rainbow colors).

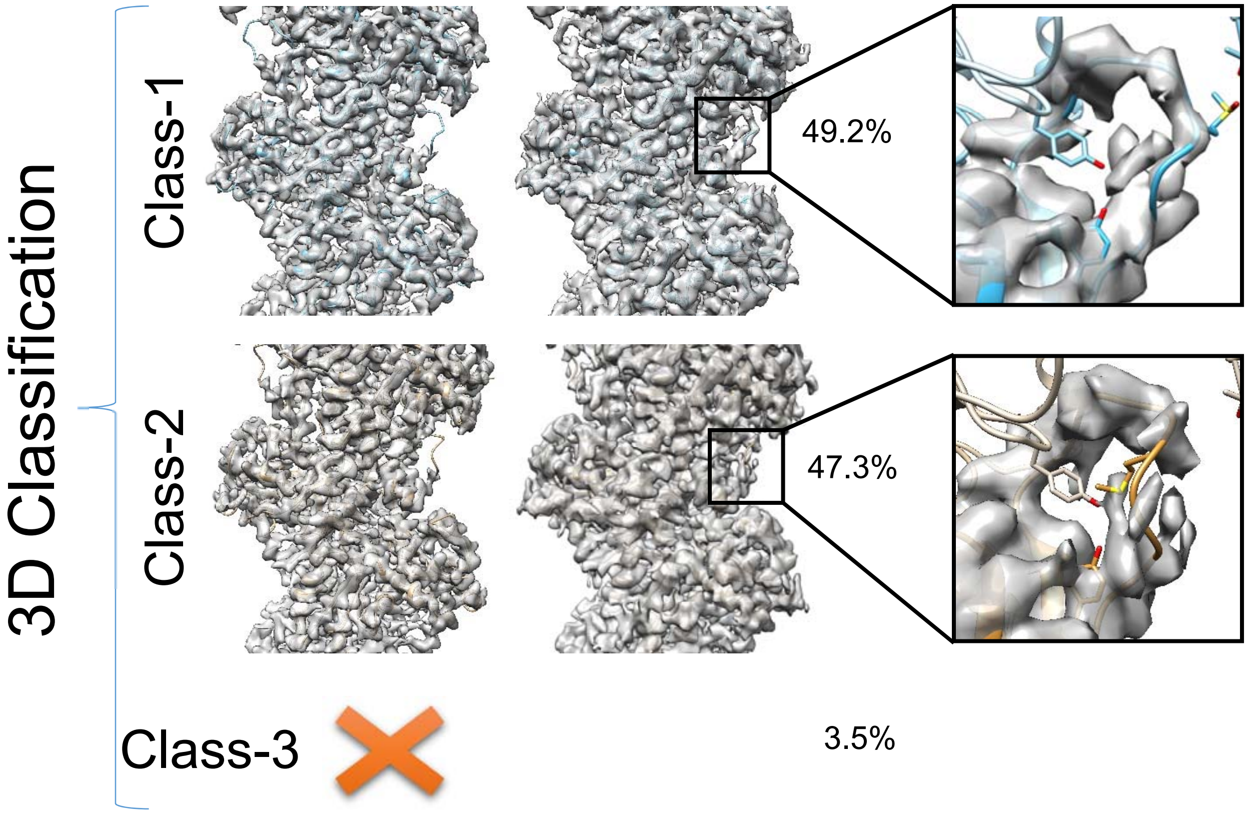

Supplementary Figure 5. **The 3D classification of Mox-F-actin.** CryoEM maps (grey shaded surface) calculated from particles of Class-1 (superimposed with cyan ribbons for its atomic model) and Class-2 of Mox-F-actin (superimposed with tan ribbon models) respectively. Each map is shown in two density thresholds. The partition of the classes are stated on the right side of the map displays. Insets on the right show zoomed views of the D-loop regions. For clarity, only one (lower) subunit in each panel is shown in grey shaded surface.

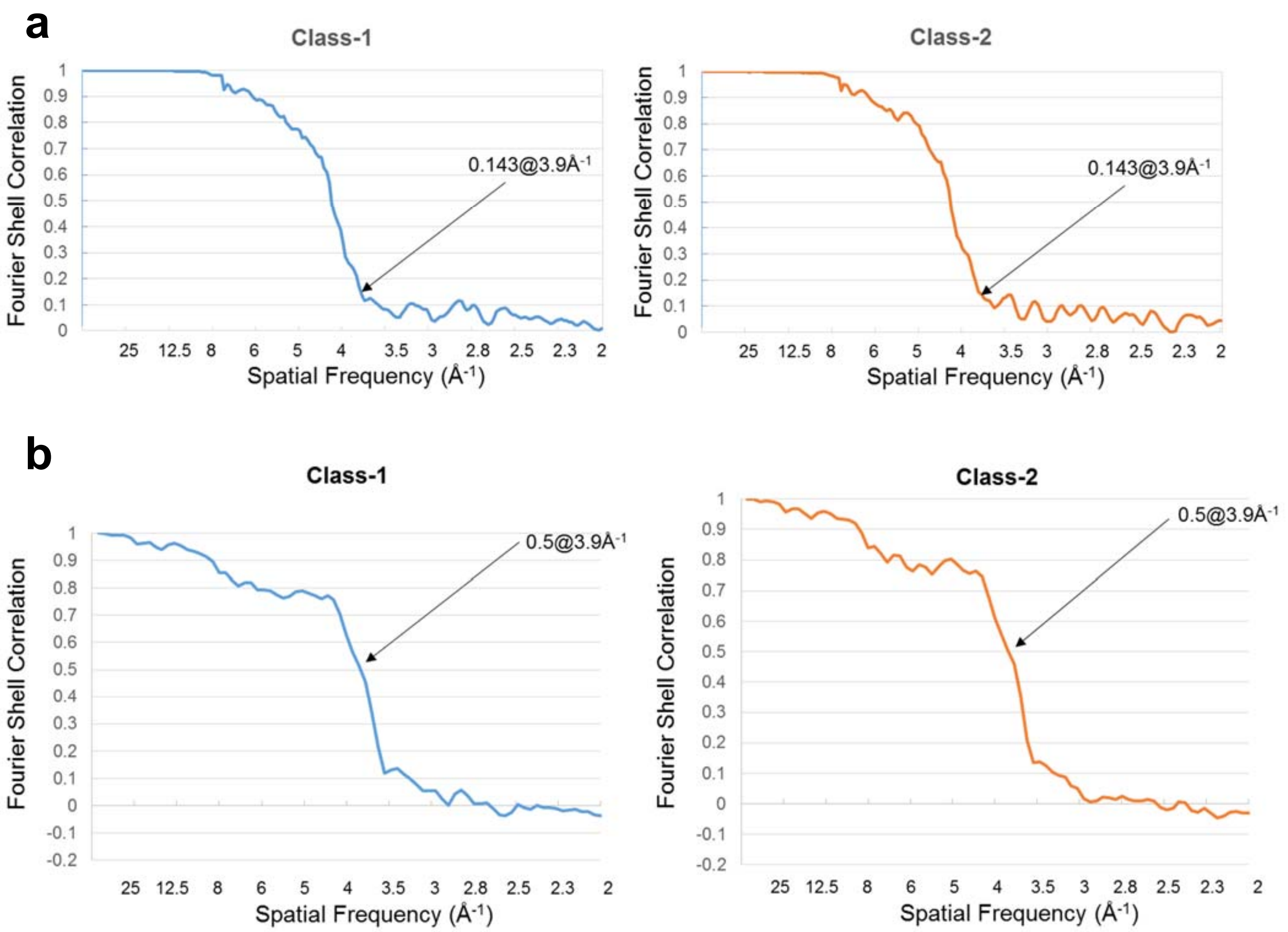

Supplementary Figure 6. **Resolution estimation of the final structures for both classes of Mox-F-actin. (a)** Fourier shell correlation (FSC) curve for the two classes of Mox-F-actin. In both classes, the FSC between two independently refined half datasets drops to 0.143 at a spatial frequency of 1/3.9 Å. **(b)** Fourier shell correlation curve for the two classes of Mox-F-actin. In both classes, the FSC between the map and its corresponding atomic model drops to 0.5 at a spatial frequency of 1/3.9 Å.

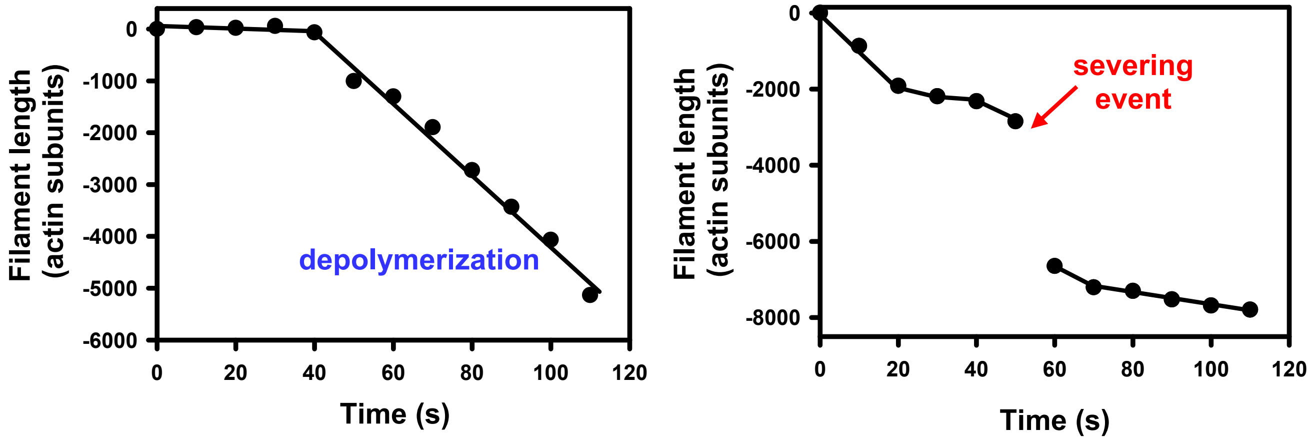

Supplementary Figure 7. **Analysis of 5C Drosophila actins disassembly after on-slide oxidation by Mical/NADPH**. As expected, we observed a moderate F-actin severing upon Mical/NADPH addition[1,2]. Note that our analysis clearly distinguishes between actin depolymerization (left) and severing events (right). Examples with Mical-oxidized WT 5C Drosophila actin are shown.

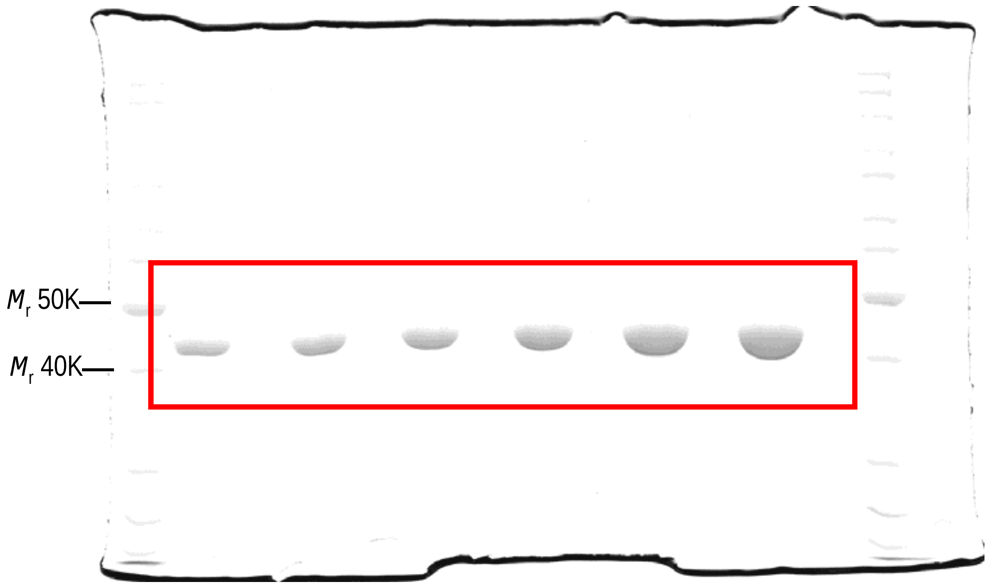

Supplementary Figure 8. Uncropped gel for Figure 1f (red box).

Supplementary Table 1. Crystallographic data and refinement statistics of Mical-oxidized G-actin (Mox-G-actin) complexed with gelsolin segment 1.

	Mox-G-actin+gelsolin segment 1*
**Data collection**	
Space group	$P2_12_12_1$
Cell dimensions	
a, *b*, *c* (Å)	56.2, 69.3, 179.2
α, β, γ (°)	90.0, 90.0, 90.0
Resolution (Å)	2.75 (2.91-2.75)**
R_{merge}	0.156 (0.528)
$I / \sigma I$	13.9 (5.2)
$CC_{1/2}$	0.999 (0.976)
Completeness (%)	100.0 (100.0)
Redundancy	20.3 (20.5)
**Refinement**	
Resolution (Å)	2.75
No. reflections	18896
R_{work} / R_{free}	0.187 / 0.199
No. atoms	
Protein	3843
Ligand/ion	42
Water	134
B-factors (Å^2)	
Protein	81.2
Ligand/ion	64.4
Water	73.1
R.m.s. deviations	
Bond lengths (Å)	0.010
Bond angles (°)	1.0
PDB ID code	5UBO

*Two crystals were merged to obtain this data set.
**Values in parentheses are for highest-resolution shell.

Supplementary Table 2. Intra- and intermolecular interactions in Mox-F-actin compared to canonical actin model (PDB: 5JLF)[3,4]

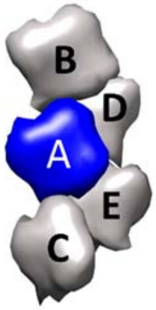

Canonical (PDB: 5JLF)		Mox-F-actin Class-1	Mox-F-actin Class-2
**Chain A**	Interaction		
**Arg37**	**A**: Asp80, Asp81		
**Arg39**	**B**: Asp286		
	**D**: Glu270		
**Asp51**	**A**: Arg37	Distance increase by 5 Å	Distance increase by 3 Å
	**A**: Lys84	Distance increase by 2 Å	
**Tyr53**	**A**: Lys61		
	**B**: Glu167		
**Arg62**	**B**: Asp288		
**Tyr69**	**A**: Arg183	Slightly different rotamers, π interaction still valid	
**His173**	**D**: Ile267, Gly268		
**Glu195**	**D**: Lys113		
**Asp244**	**B**: Arg290		
**Gly42-Gly48**	B: SD3	Oxidized M44, side chain outside the pocket	Oxidized M44 but good fit to pocket
**Pro38-Lys50**	**B**: Tyr169	Interaction between M47 and Tyr169 abolished, hydrogen bond between M47-O and T351	Hydrogen bond between M47-O and Tyr169

Supplementary References

1. Grintsevich, E. E. *et al.* F-actin dismantling through a redox-driven synergy between Mical and cofilin. *Nat Cell Biol* **18,** 876–885 (2016).

2. Hung, R. J., Pak, C. W. & Terman, J. R. Direct redox regulation of F-actin assembly and disassembly by Mical. *Science* **334,** 1710–1713 (2011).

3. Von der Ecken, J., Heissler, S. M., Pathan-Chhatbar, S., Manstein, D. J. & Raunser, S. Cryo-EM structure of a human cytoplasmic actomyosin complex at near-atomic resolution. *Nature* **534,** 724–728 (2016).

4. Von der Ecken, J. *et al.* Structure of the F-actin-tropomyosin complex. *Nature* **519,** 114–117 (2015).
