# Supplementary material for: Catastrophic disassembly of actin filaments via Mical-mediated oxidation
Source: Nat Commun. 2017 Dec 19;8:2183. doi: 10.1038/s41467-017-02357-8 (PMC5736627; doi:10.1038/s41467-017-02357-8)
Supplement: Supplementary file 7 — Description of Additional Supplementary Files [file 41467_2017_2357_MOESM7_ESM.pdf]

Description of Additional Supplementary Files

File Name: Supplementary Movie 1
Description: Depolymerization of unoxidized F-actin upon monomers wash off. Bar=10µm. Movie speed: 3 frames/s

File Name: Supplementary Movie 2
Description: Depolymerization of Mox-F-actin upon monomers wash off. Bar=10µm. Movie speed: 3 frames/s

File Name: Supplementary Movie 3
Description: Binding of M44-O in the hydrophobic cleft of Mox-F-actin Class-1. Panel is rotated along X axis for 40 degrees up and down, showing the three dimensional relationships of M44-O side chain in respect to its binding pocket in the hydrophobic cleft of Mox-actin Class-1.

File Name: Supplementary Movie 4
Description: Binding of M44-O in the hydrophobic cleft of Mox-F-actin Class-2. Panel is rotated along X axis for 40 degrees up and down, showing the three dimensional relationships of M44-O side chain in respect to its binding pocket in the hydrophobic cleft of Mox-actin Class-2. Movie 5.

File Name: Supplementary Movie 5
Description: Binding of unoxidized M44 in the hydrophobic cleft of F-actin. Panel is rotated along X axis for 40 degrees up and down, showing the three dimensional relationships of M44 side chain in respect to its binding pocket in the hydrophobic cleft of F-actin (PDB#: 5JLF).
